# Dynamic hemoglobin trajectories and mortality in hemodialysis patients: a joint modeling study with iron and phosphorus markers

**DOI:** 10.3389/fmed.2026.1770595

**Published:** 2026-03-25

**Authors:** Min-Jia Li, Hong-Yong Liu, Yun-Qiang Zhang, Jian-Hong Zhang, Sheng-Rong Li, Ya Zhu, Xiu-Lan Yang, Yu Zeng

**Affiliations:** Department of Nephrology, The Third Affiliated Hospital of Sun Yat-Sen University, Yuedong Hospital, Meizhou, Guangdong, China

**Keywords:** dynamic prediction, ferritin, hemodialysis, hemoglobin, joint model, longitudinal biomarker, mortality, phosphorus

## Abstract

**Background:**

Mortality remains high among maintenance hemodialysis (MHD) patients, and laboratory biomarkers such as hemoglobin, ferritin, and phosphorus exhibit substantial temporal variability. Static measurements may fail to adequately reflect dynamic prognostic changes.

**Objective:**

This study evaluated the longitudinal trajectories of hemoglobin, log-transformed ferritin, and phosphorus, and assessed their dynamic associations with all-cause mortality in MHD patients using joint modeling.

**Methods:**

A total of 254 MHD patients with 814 repeated measurements were analyzed. Linear mixed-effects models were linked with Cox proportional hazards models. Three joint model structures were compared using information criteria, and dynamic predictive performance was evaluated using time-dependent AUC and Brier scores.

**Results:**

Over a median 18-month follow-up, 62 deaths (24.4%) occurred. Higher hemoglobin levels were independently associated with lower mortality (per 10 g/L increase: HR = 0.97; 95% CI: 0.95–1.00). Log-ferritin showed a negative but nonsignificant association, while phosphorus did not improve model fit. The hemoglobin + log-ferritin model achieved the best discrimination (AUC ≈ 0.70–0.75). Sensitivity analyses incorporating phosphorus or replacing ferritin with transferrin saturation produced consistent results, confirming robustness.

**Conclusion:**

Dynamic hemoglobin changes show a modest but consistent inverse association with mortality risk in MHD patients and provide stable predictive value. Joint modeling may enhance risk stratification and support individualized anemia management in hemodialysis.

## 1 Introduction

Despite substantial advances in dialysis therapy, patients on maintenance hemodialysis (MHD) continue to experience markedly elevated mortality compared with the general population, and large variation exists across countries and regions (1). For example, among prevalent HD patients in Japan, Europe, and the United States, the crude 1-year mortality has been reported to be approximately 6.6, 15.6, and 21.7%, respectively (2). In contrast, recent Chinese data from a large-scale cohort (Beijing, 2014–2020; 24,363 MHD patients) show an annual mortality rate ranging around 7.4–8.0% (3). Although differences in patient demographics, dialysis practice, and healthcare resources preclude a simple global estimation, the persistently elevated mortality burden among MHD patients underscores the need for improved mortality risk stratification, individualized monitoring, and tailored treatment decisions.

Hemoglobin, ferritin, and phosphorus are key laboratory markers closely related to anemia management, iron metabolism, and mineral bone disorders in MHD patients (4, 5). Current clinical guidelines highlight maintaining hemoglobin within an optimal range and controlling phosphorus levels to prevent cardiovascular complications (6, 7). However, these biomarkers exhibit considerable intra- and inter-patient variability due to inflammation, nutritional status, treatment fluctuations, and changes in residual kidney function (8, 9). Thus, isolated or baseline measurements cannot fully reflect their dynamic prognostic relationships with mortality.

Most existing studies have relied on baseline values, time-averaged measurements, or simple longitudinal changes, which fail to account for continuous measurement error and informative dropout due to death (10). Furthermore, the prognostic implications of iron metabolism biomarkers remain inconsistent: elevated ferritin may reflect either adequate iron stores or inflammation, whereas transferrin saturation (TSAT) is susceptible to short-term fluctuation and analytical bias (11, 12). Evidence on the dynamic effect of serum phosphorus on mortality also remains limited and controversial (13, 14). Critically, there is a paucity of research adopting joint modeling approaches to evaluate these time-varying biomarkers in real-world Chinese hemodialysis populations.

Joint models (JM), integrating linear mixed-effects models with survival analysis, provide a rigorous analytical solution by capturing individual biomarker trajectories, accounting for time-varying measurement error, and modeling the association between longitudinal processes and mortality risk (15). Importantly, JM enable dynamic and individualized survival prediction that holds critical clinical value for dialysis patient management. Therefore, this study aimed to characterize the longitudinal trajectories of hemoglobin, log-transformed ferritin, and phosphorus in MHD patients, to assess their dynamic associations with all-cause mortality using joint models, and to compare predictive performance across different biomarker combinations. Findings from this real-world Chinese hemodialysis cohort may contribute to improving risk stratification and guiding personalized monitoring strategies in clinical practice.

## 2 Methodology

### 2.1 Study design and participants

**Figure 1:**
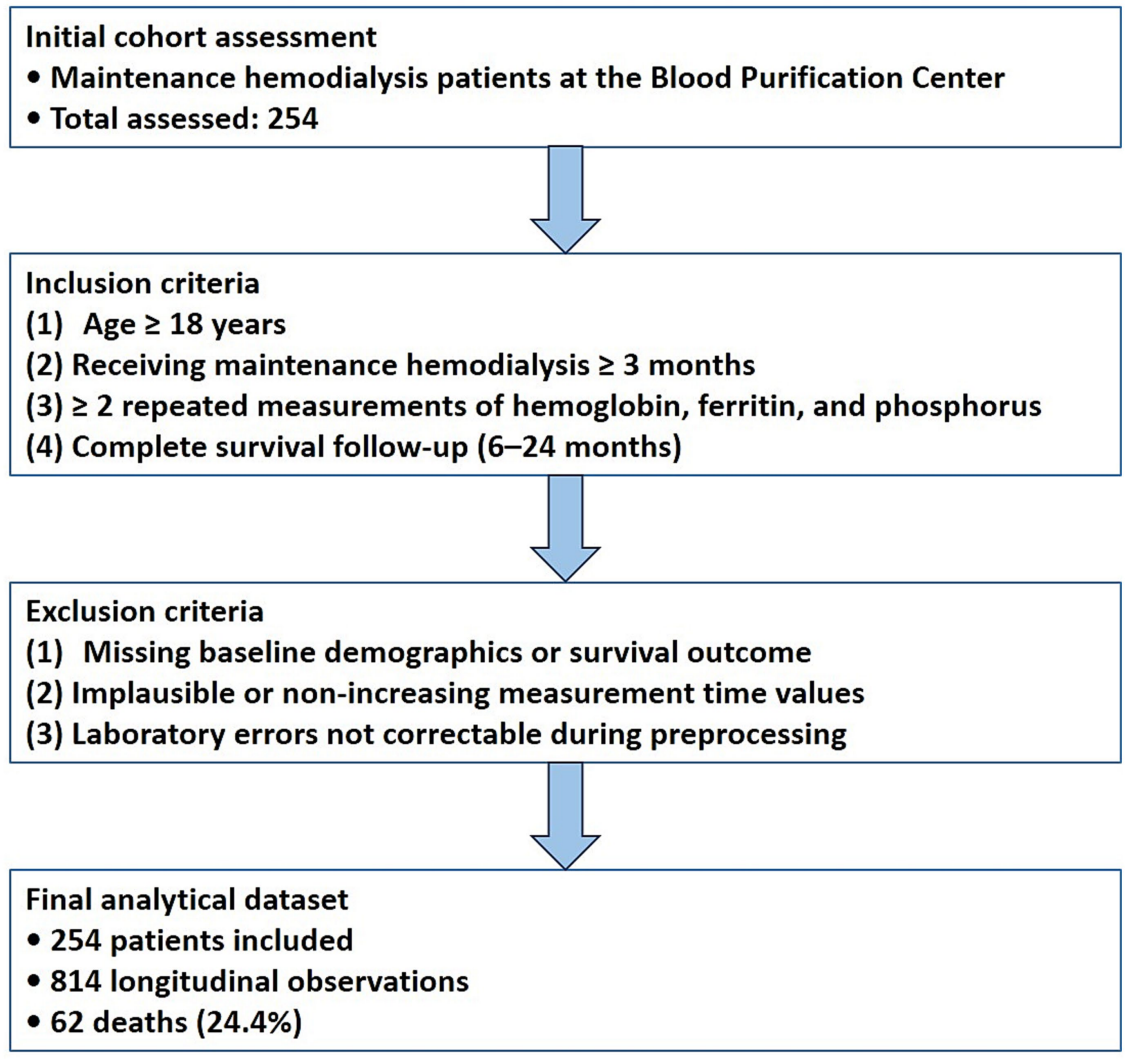
Study flow diagram. Flow of cohort selection, longitudinal laboratory measurements, and inclusion in joint modeling and internal dynamic prediction evaluation.

This study was a retrospective cohort analysis of maintenance hemodialysis patients at the Blood Purification Center of the Third Affiliated Hospital of Sun Yat-sen University, Yuedong Hospital. Patients who received stable hemodialysis and had at least two repeated laboratory measurements were eligible. Individuals were excluded if longitudinal records were incomplete or follow-up information was missing. The flow of cohort selection, application of inclusion and exclusion criteria, and construction of the final analytical dataset is shown in Figure 1.

### 2.2 Data collection and variables

Baseline demographic and clinical data, including age (years), sex, and diabetes mellitus status, were extracted from electronic medical records. In addition, routinely monitored laboratory variables at the start of follow-up comprised albumin (g/L), hemoglobin (g/L), serum iron (μmol/L), total iron-binding capacity (TIBC, μmol/L), transferrin saturation (TSAT, %), parathyroid hormone (PTH, pg./mL), serum calcium (mmol/L), serum phosphorus (mmol/L), blood urea nitrogen (BUN, mmol/L), serum creatinine (μmol/L), serum uric acid (μmol/L), total cholesterol (mmol/L), and low-density lipoprotein cholesterol (LDL-C, mmol/L).

Transferrin saturation (TSAT) was calculated as serum iron divided by total iron-binding capacity multiplied by 100:


TSAT(%)=(serum iron/TIBC)×100.

All laboratory tests, including hemoglobin, serum ferritin (ng/mL), and serum phosphorus, were typically measured approximately every three months as part of routine hemodialysis care. In the joint models, hemoglobin, log-transformed ferritin, and phosphorus were treated as the key longitudinal biomarkers. Because ferritin showed a markedly right-skewed distribution, its values were natural log-transformed before modeling to improve the normality of residuals.

### 2.3 Outcome definition

The primary outcome was all-cause mortality. Follow-up time was defined as the number of months from the start of longitudinal observation (baseline visit) to the occurrence of death or censoring. For each patient, follow-up ended at the date of death or at the last available follow-up visit if death did not occur. Patients without death events were censored at their last recorded follow-up.

### 2.4 Longitudinal submodels

Each laboratory biomarker was modeled using linear mixed-effects models (LME) to account for within-individual correlation and irregular measurement intervals.

1. Hemoglobin: random intercept and random slope.
2. Log-Ferritin: random intercept.
3. Phosphorus: random intercept.

Time scale for all longitudinal submodels was Follow_month (months since dialysis initiation). The LME models were fitted using the nlme package in R.

### 2.5 Survival submodel

A Cox proportional hazards model was used to estimate the association between baseline covariates and mortality. The model included age, sex, and diabetes as fixed effects. The baseline Cox model (cox_A) was fitted using the survival package, and the model matrix was retained for downstream joint modeling.

### 2.6 Joint modeling approach

To simultaneously evaluate longitudinal biomarker trajectories and mortality risk, joint models for longitudinal and time-to-event data were fitted using the JMbayes2 package, adopting the “current value” association structure.

The longitudinal submodel for each biomarker k and patient i at time t was specified using a linear mixed-effects model: $y_{\mathrm{ik}}(t)=x_{\mathrm{ik}}{\left(t\right)}^{⊤}\beta_{k}+z_{\mathrm{ik}}{\left(t\right)}^{⊤}b_{\mathrm{ik}}+\varepsilon_{\mathrm{ik}}(t),$ where $y_{\mathrm{ik}}(t)$ is the observed biomarker value, $\beta_{k}$ fixed effects, $b_{\mathrm{ik}}$ patient-specific random effects, and $\varepsilon_{\mathrm{ik}}(t)$ measurement error.

The time-to-event process was modeled using a proportional hazards structure: $h_{i}(t)=h_{0}(t)\exp(\gamma^{⊤}X_{i}+\alpha \;m_{i}(t)),$ where $h_{i}(t)$ is the hazard of death, $X_{i}$ baseline covariates (age, sex, diabetes), $m_{i}(t)$ the predicted “true” biomarker trajectory from the longitudinal submodel, and α the association parameter linking the longitudinal biomarker with mortality risk.

Three prespecified models were fitted:

1. Model A (main model): Hemoglobin + log-Ferritin.
2. Model A + (extended model): Hemoglobin + log-Ferritin + Phosphorus.
3. Model B (iron-related sensitivity model): Hemoglobin + TSAT.

All models used Follow_month as the internal time scale. Posterior distributions were obtained using Markov chain Monte Carlo (MCMC) with three chains, 6,500 iterations per chain, a burn-in of 500 iterations, and thinning of 1. Convergence was evaluated with trace plots and the Gelman–Rubin statistic (R-hat).

The modeling framework followed established methodology for joint models of longitudinal and survival data (16–18).

### 2.7 Model comparison and predictive performance

Model fit was evaluated using Information Criteria, including the deviance information criterion (DIC), the widely applicable information criterion (WAIC), and the log pseudomarginal likelihood (LPML). To assess dynamic prediction accuracy, we computed time-dependent area under the curve (AUC) and time-dependent Brier scores using the dynamic prediction functions of JMbayes2. Landmark times were set at 6 and 12 months after dialysis initiation, and we evaluated prediction of all-cause mortality within the subsequent 6 and 12 months (fixed prediction horizons).

### 2.8 Sensitivity analyses

Two independent sensitivity analyses were performed:

1. Replacing log-Ferritin with transferrin saturation (TSAT), and.
2. Additionally including phosphorus in the longitudinal component.

All sensitivity models retained the same survival submodel and association structure.

### 2.9 Software

All analyses were conducted in R version 4.3.2 using the following packages: data.table, nlme, survival, JMbayes2, ggplot2, and dplyr.

### 2.10 Ethical approval

The study complied with the Declaration of Helsinki and was approved by the Ethics Committee of The Third Affiliated Hospital of Sun Yat-sen University, Yuedong Hospital. Patient data were anonymized prior to analysis.

## 3 Results

### 3.1 Baseline characteristics

**Figure 2:**
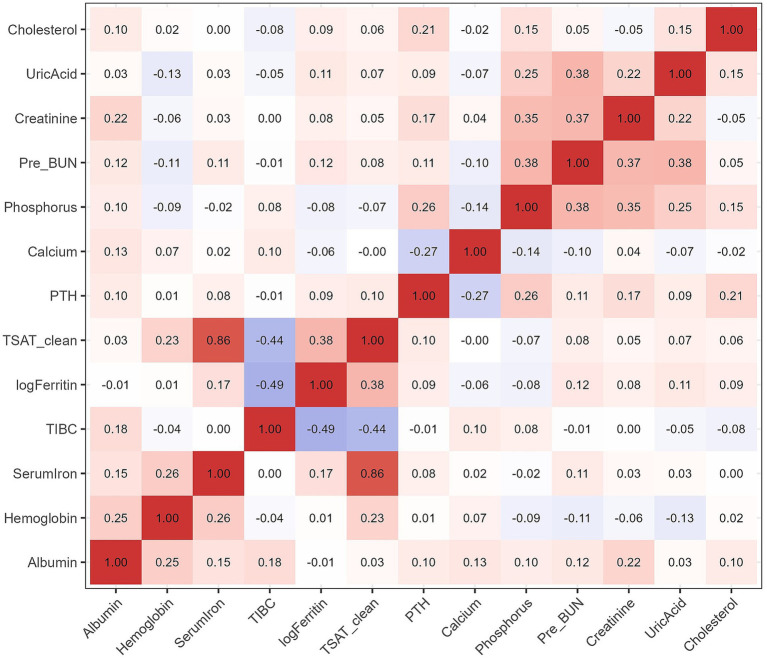
Baseline correlation heatmap. Pairwise correlations among baseline laboratory biomarkers (hemoglobin, ferritin, phosphorus, albumin, serum iron, TSAT, and PTH). This analysis helped assess potential collinearity prior to model specification.

A total of 254 patients receiving maintenance hemodialysis contributed 814 longitudinal measurements over a median follow-up of 18 months, during which 62 deaths occurred (24.4%). The median age was 60.0 years and 34.3% were female (Table 1). Patients who died were older and had lower albumin levels at baseline compared with survivors, while other variables showed no significant group differences. Correlations among baseline biomarkers are displayed in the heatmap (Figure 2), without evidence of strong multicollinearity. The distribution of baseline hemoglobin according to patient outcome is shown in Supplementary Figure S1.

**Table 1:** Baseline characteristics of maintenance hemodialysis patients by survival status.

Variable	Overall	Non-death	Death	*P*_value	ASD
Age (years)	60.00 [50.25–69.00]	58.00 [49.00–65.00]	68.50 [59.00–72.75]	<0.001	0.746
Male sex, n (%)	167 (65.7)	152 (79.2)	15 (24.2)	0.077	0.29
Diabetes mellitus, n (%)	94 (37.0)	67 (34.9)	27 (43.5)	0.282	0.177
Albumin (g/L)	36.40 [34.10–38.50]	36.70 [34.40–38.60]	35.45 [32.15–37.77]	0.004	0.396
Hemoglobin (g/L)	92.50 [79.00–106.00]	93.50 [79.00–107.00]	89.50 [76.75–101.75]	0.202	0.26
Serum iron (μmol/L)	8.65 [6.90–11.60]	8.60 [6.77–12.53]	8.75 [7.50–10.28]	0.975	0.05
Total iron-binding capacity (TIBC, μmol/L)	44.05 [38.23–50.77]	44.35 [39.20–50.97]	41.85 [36.15–49.50]	0.114	0.209
Ferritin (ng/mL), log-transformed in analysis	113.70 [51.78–213.10]	115.95 [51.23–201.78]	97.35 [52.55–223.10]	0.832	0.003
Transferrin saturation (TSAT, %)	20.35 [15.03–28.14]	20.22 [14.29–28.37]	21.43 [16.49–27.18]	0.381	0.069
Parathyroid hormone (PTH, pg./mL)	289.15 [174.07–455.20]	286.85 [171.90–470.50]	301.40 [187.42–426.43]	0.987	0.148
Calcium (mmol/L)	2.16 [1.99–2.31]	2.17 [2.01–2.33]	2.13 [1.96–2.26]	0.232	0.171
Phosphorus (mmol/L)	1.75 [1.40–2.15]	1.70 [1.34–2.16]	1.81 [1.49–2.08]	0.505	0.042
Pre-dialysis BUN (mmol/L)	23.89 [20.26–28.90]	24.56 [20.74–29.02]	22.48 [19.50–27.92]	0.07	0.26
Creatinine (μmol/L)	822.35 [680.90–1036.88]	823.65 [679.78–1041.35]	792.65 [686.15–983.75]	0.378	0.099
Uric acid (μmol/L)	484.80 [416.23–548.50]	489.05 [410.00–555.25]	461.25 [422.00–537.75]	0.664	0.06
Total cholesterol (mmol/L)	4.21 [3.42–4.90]	4.25 [3.43–4.89]	4.13 [3.27–5.24]	0.87	0.04
LDL-C (mmol/L)	2.08 [1.53–2.57]	2.11 [1.55–2.50]	1.94 [1.52–2.84]	0.998	0.054

Data are presented as median [IQR] for continuous variables and n (%) for categorical variables.

Group comparisons were performed using Wilcoxon rank-sum tests for continuous variables and χ^2^ tests for categorical variables.

BUN, blood urea nitrogen; LDL-C, low-density lipoprotein cholesterol; TSAT, transferrin saturation.

PTH, parathyroid hormone.

### 3.2 Univariable joint model analysis

In univariable joint models, lower hemoglobin was significantly associated with increased mortality risk (per 10 g/L decrease: HR 0.97; 95% CI 0.95–1.00; *p* = 0.037). Log-transformed ferritin showed a nonsignificant inverse association (HR 0.76; 95% CI 0.52–1.34; *p* = 0.21). Serum phosphorus demonstrated a positive but nonsignificant association with mortality (HR 1.73; 95% CI 0.92–2.98; *p* = 0.11). TSAT and serum iron were not associated with mortality. Full results are provided in Supplementary Table S2.

### 3.3 Longitudinal biomarker trajectories

**Figure 3:**
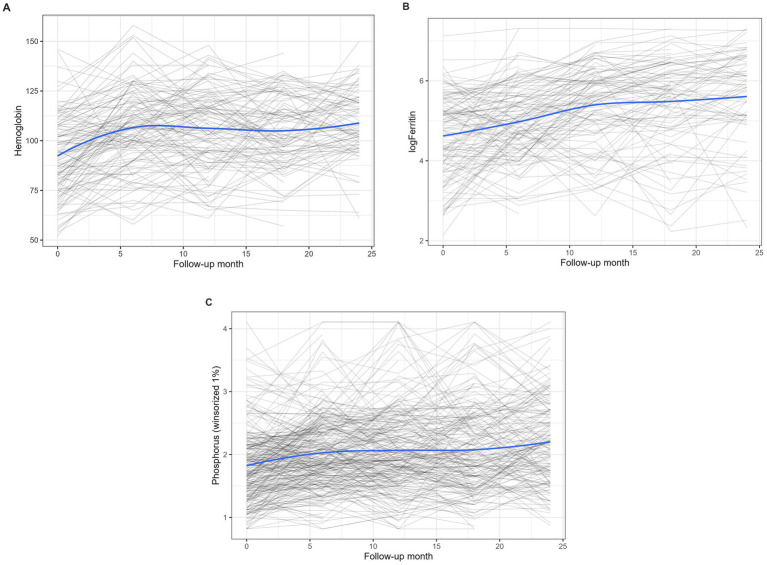
Predicted longitudinal biomarker trajectories. Fitted trajectories derived from linear mixed-effects models with subject-specific random effects: **(A)** Hemoglobin, **(B)** log (Ferritin), **(C)** Phosphorus.

Three biomarker trajectories are shown in Figure 3, while additional visualizations of hemoglobin longitudinal patterns are presented in Supplementary Figures S2, S3. Hemoglobin remained relatively stable over follow-up, whereas log-ferritin gradually increased and phosphorus fluctuated considerably. These heterogeneous temporal patterns support the need for a joint modeling framework to appropriately capture longitudinal risk information.

### 3.4 Main joint model: hemoglobin + log-ferritin (model a)

**Figure 4:**
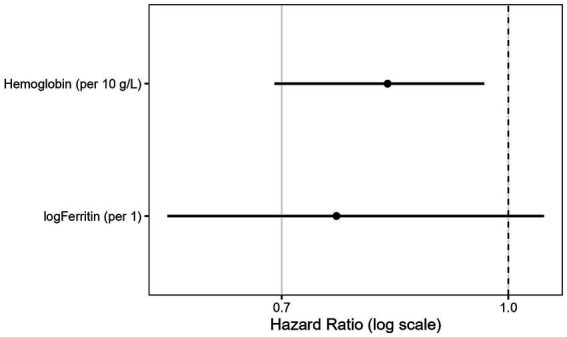
Joint model effects for the main model (hemoglobin + logFerritin). Hazard ratios (HRs) and 95% confidence intervals for the association between longitudinal biomarker values and mortality, estimated from the joint model (model A).

**Figure 5:**
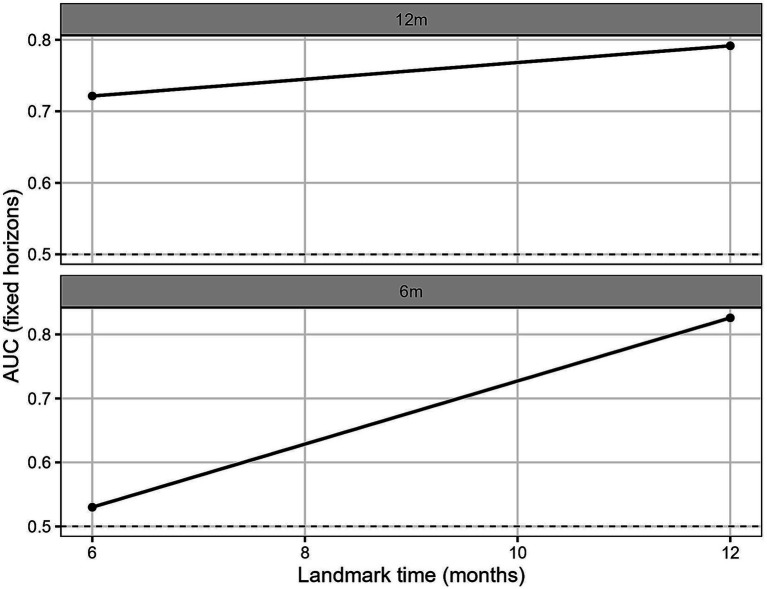
Dynamic AUC for internal validation. Time-dependent AUC at landmark times of 6 and 12 months, predicting mortality within the subsequent 6 or 12 months, based on joint model internal cross-validation.

**Figure 6:**
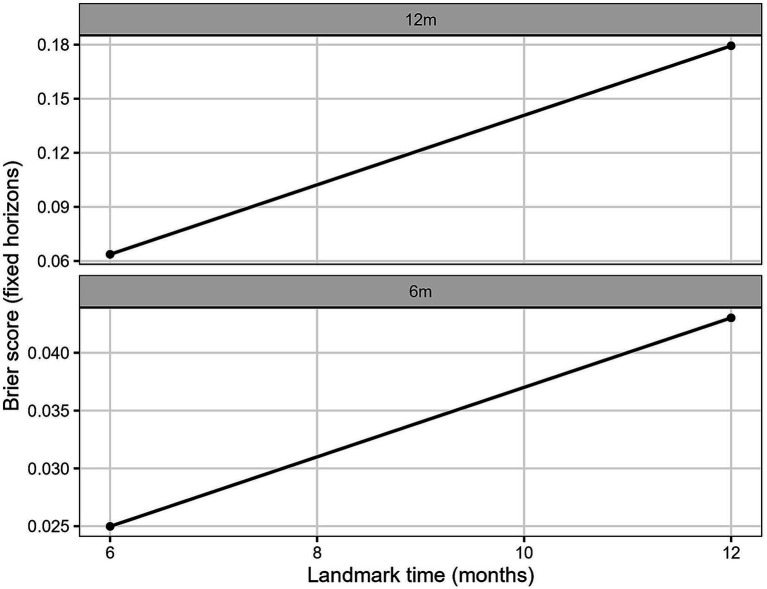
Dynamic Brier score for internal validation. Prediction error curves (Brier score) for 6- and 12-month prediction horizons in internal validation.

In the multivariable joint model, hemoglobin retained an independently protective association with mortality (per 10 g/L: HR 0.97; 95% CI 0.95–1.00; *p* = 0.038), while log-ferritin remained nonsignificant. The forest plot of association parameters is presented in Figure 4. The model showed good internal predictive performance, with time-dependent AUC values consistently around 0.70–0.75 for 6- and 12-month prediction horizons (Figure 5), and low prediction error according to dynamic Brier scores (Figure 6 and Table 2).

**Table 2:** Internal dynamic prediction accuracy.

Horizon	Tstart	*n*	iAUC	iBrier
12 m	6	234	0.72133	0.063701
12 m	12	222	0.791516	0.179369
6 m	6	234	0.529971	0.024984
6 m	12	222	0.825943	0.043016

Internal validation metrics including dynamic AUC and Brier score for 6- and 12-month prediction horizons.

### 3.5 Extended model with phosphorus (model a+)

Adding phosphorus did not improve model fit or predictive performance (higher WAIC and DIC), and phosphorus remained nonsignificant (HR ≈ 2.01; *p* ≈ 0.10), indicating that its inclusion provided limited additional prognostic value (Table 3).

**Table 3:** Model comparison (IC values).

Model	Longitudinal biomarkers	DIC (marginal)	DIC (conditional)	WAIC (marginal)	WAIC (conditional)	LPML (marginal)	LPML (conditional)
Model A	Hemoglobin + logFerritin	−11606.233	−8518.854	2,355,298	2,358,233	−4917.138	−6425.719
Model A+	Hemoglobin + logFerritin + Phosphorus	−3030.171	192.497	1,118,911	1,118,700	−6931.807	−8238.013

Comparison of joint models using DIC, WAIC, and LPML for: Model A (Hb + logFerritin); Model A + (Hb + logFerritin + Phosphorus); Model B (Hb + TSAT).

### 3.6 Sensitivity model with TSAT (model B)

Replacing log-ferritin with TSAT led to substantially poorer model performance and uncertain estimates, with no significant association between TSAT and mortality (HR ≈ 1.82; *p* = 0.59; Table 3).

### 3.7 Sensitivity analyses

All sensitivity analyses demonstrated consistent effect directions and reinforced hemoglobin as the most informative biomarker for dynamic mortality prediction in hemodialysis patients. Detailed estimates are provided in Supplementary Table S3 and Supplementary Figure S4.

## 4 Discussion

In this longitudinal cohort of maintenance hemodialysis patients, we found that dynamic hemoglobin levels were modestly yet independently associated with mortality risk, whereas log-transformed ferritin showed a negative but non-significant association. Joint models that incorporated repeated measurements captured prognostic information beyond single baseline assessments and achieved good overall discrimination. Among the evaluated combinations, the model including hemoglobin and log-transformed ferritin achieved the best overall performance, while phosphorus and TSAT did not materially enhance predictive ability.

The observed associations reflect key physiological mechanisms in hemodialysis patients. Declines in hemoglobin may reflect inadequate erythropoiesis, chronic inflammation, or malnutrition, all of which contribute to adverse outcomes (19). Ferritin, despite its known variability, captures the interplay between iron storage, inflammation, and ESA resistance, and its longitudinal pattern appears more informative than single measurements (20). The weak and imprecise estimates for phosphorus and TSAT suggest that short-term fluctuations in mineral metabolism or iron availability may not translate into stable risk signals when evaluated alongside stronger hematologic markers. Phosphorus levels in hemodialysis patients are influenced by multiple factors including dietary intake, dialysis clearance, and phosphate binder therapy, while TSAT is highly responsive to iron supplementation and ESA therapy, which may attenuate stable longitudinal associations with mortality. These findings support incorporating biomarker dynamics, rather than isolated values, into mortality risk assessment.

Our results align with prior studies that identified time-varying hemoglobin trajectories as prognostic indicators in dialysis populations (21, 22). Similar findings have been reported in analyses of repeated ferritin or CRP levels in chronic disease cohorts, where joint modeling approaches captured prognostic information that traditional Cox models could not (23). Previous work in diabetes, heart failure, and oncology has likewise demonstrated that longitudinal biomarkers better reflect disease activity, treatment response, and patient heterogeneity (24, 25). The consistency across disease domains reinforces the suitability of joint models for conditions characterized by frequent laboratory monitoring and dynamic physiological changes.

This study leveraged joint modeling to link longitudinal processes and survival outcomes, allowing simultaneous estimation while accounting for measurement error, irregular follow-up times, and intra-individual correlation (26). Dynamic prediction provided individualized risk estimates at each time point, demonstrating favorable discrimination and calibration in internal validation. By evaluating several biomarker combinations and conducting predefined sensitivity analyses, we ensured robustness across alternative parameterizations and data preprocessing strategies. Compared with approaches using static baseline values, the present framework more accurately reflects the evolving clinical state of hemodialysis patients.

The findings highlight the potential value of integrating repeated hemoglobin and ferritin measurements into routine risk stratification. Dynamic monitoring may help clinicians identify patients at elevated risk earlier and refine anemia management strategies, including ESA dosing, iron supplementation, and evaluation for inflammation or occult blood loss. Because phosphorus and TSAT did not meaningfully improve prediction when added to the model, routine inclusion of these markers in dynamic mortality risk tools may not be necessary. The modeling approach could be incorporated into clinical decision-support systems as laboratory data accumulate over time.

Key strengths include dense longitudinal sampling, robust model structure, and comprehensive internal validation. Nonetheless, several limitations merit consideration. The study was conducted at a single center, and external generalizability remains to be established. Biomarkers such as ferritin may be influenced by acute events or measurement variability, which could introduce noise despite model adjustment. Sample size and event count were modest, limiting the precision of estimates for weaker predictors. In addition, several potentially relevant clinical variables, such as inflammatory markers, dialysis adequacy parameters, and comorbidity burden, were not available for inclusion in the models and may have influenced risk estimates. The absence of external validation also restricts the assessment of transportability to broader dialysis populations. Future studies using independent external cohorts will be necessary to confirm the generalizability of the proposed joint modeling framework.

Future work should evaluate joint models in larger multi-center cohorts and incorporate additional dynamic markers such as CRP, PTH, and nutritional indices. Exploration of non-linear trajectories, latent class structures, and machine learning–based dynamic prediction may further enhance model performance. Integration with digital dialysis platforms could enable real-time individualized risk prediction and facilitate precision medicine approaches in hemodialysis care.

## 5 Conclusion

Dynamic patterns of hemoglobin were independently associated with mortality, whereas log-transformed ferritin showed a protective trend without reaching statistical significance. Joint models provided improved characterization of prognostic information compared with static measures and support the incorporation of longitudinal biomarker information into risk stratification strategies for maintenance hemodialysis patients.

## Data Availability

The raw data supporting the conclusions of this article will be made available by the authors, without undue reservation.

## Supplementary material

SUPPLEMENTARY FIGURE S1 Baseline hemoglobin distribution by patient outcome. Baseline hemoglobin levels were compared between patients who survived and those who died during follow-up. Each point represents an individual patient at baseline. The boxes represent the interquartile range with the median line, and whiskers indicate the data range excluding outliers.

SUPPLEMENTARY FIGURE S2 Longitudinal hemoglobin trajectories by patient outcome. Mean hemoglobin levels during follow-up are shown for patients who survived and those who died. Shaded areas represent 95% confidence intervals.

SUPPLEMENTARY FIGURE S3 Individual hemoglobin trajectories by patient outcome. Grey lines represent individual patient hemoglobin trajectories. Solid lines and shaded bands indicate group mean hemoglobin levels and 95% confidence intervals over follow-up.

SUPPLEMENTARY FIGURE S4 Sensitivity analysis: forest plot of alternative joint models. Hazard ratios (HRs) for Models A+, A+_winsor, and Model B (TSAT model) demonstrating the robustness of associations across alternative model specifications.
