# Supplementary figures and images for: Dynamic hemoglobin trajectories and mortality in hemodialysis patients: a joint modeling study with iron and phosphorus markers
Source: Front Med (Lausanne). 2026 Mar 25;13:1770595. doi: 10.3389/fmed.2026.1770595 (PMC13056859; doi:10.3389/fmed.2026.1770595)

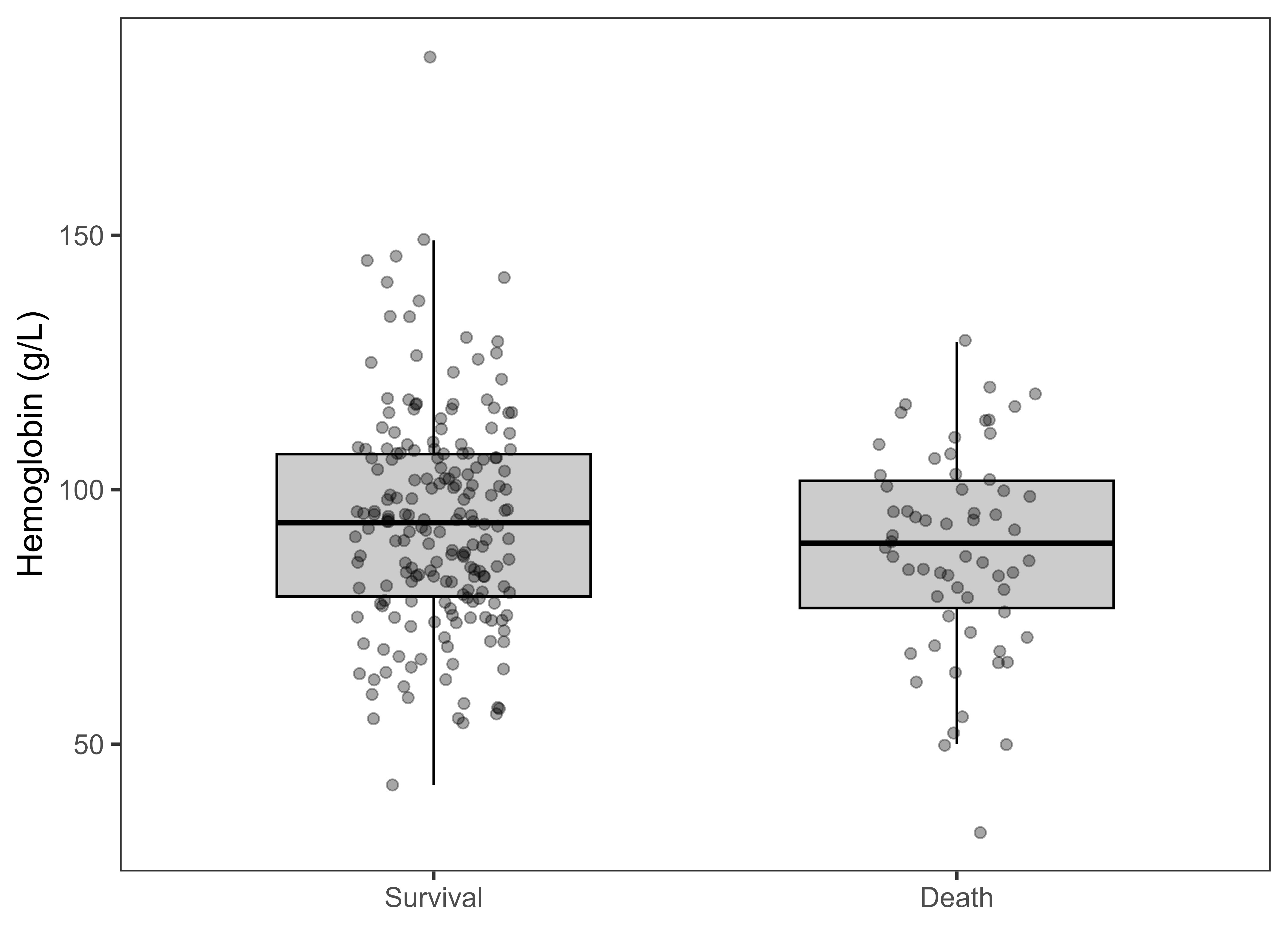

Supplement: SUPPLEMENTARY FIGURE S1 — Baseline hemoglobin distribution by patient outcome. Baseline hemoglobin levels were compared between patients who survived and those who died during follow-up. Each point represents an individual patient at baseline. The boxes represent the interquartile range with the median line, and whiskers indicate the data range excluding outliers. [file Image_1.JPEG]

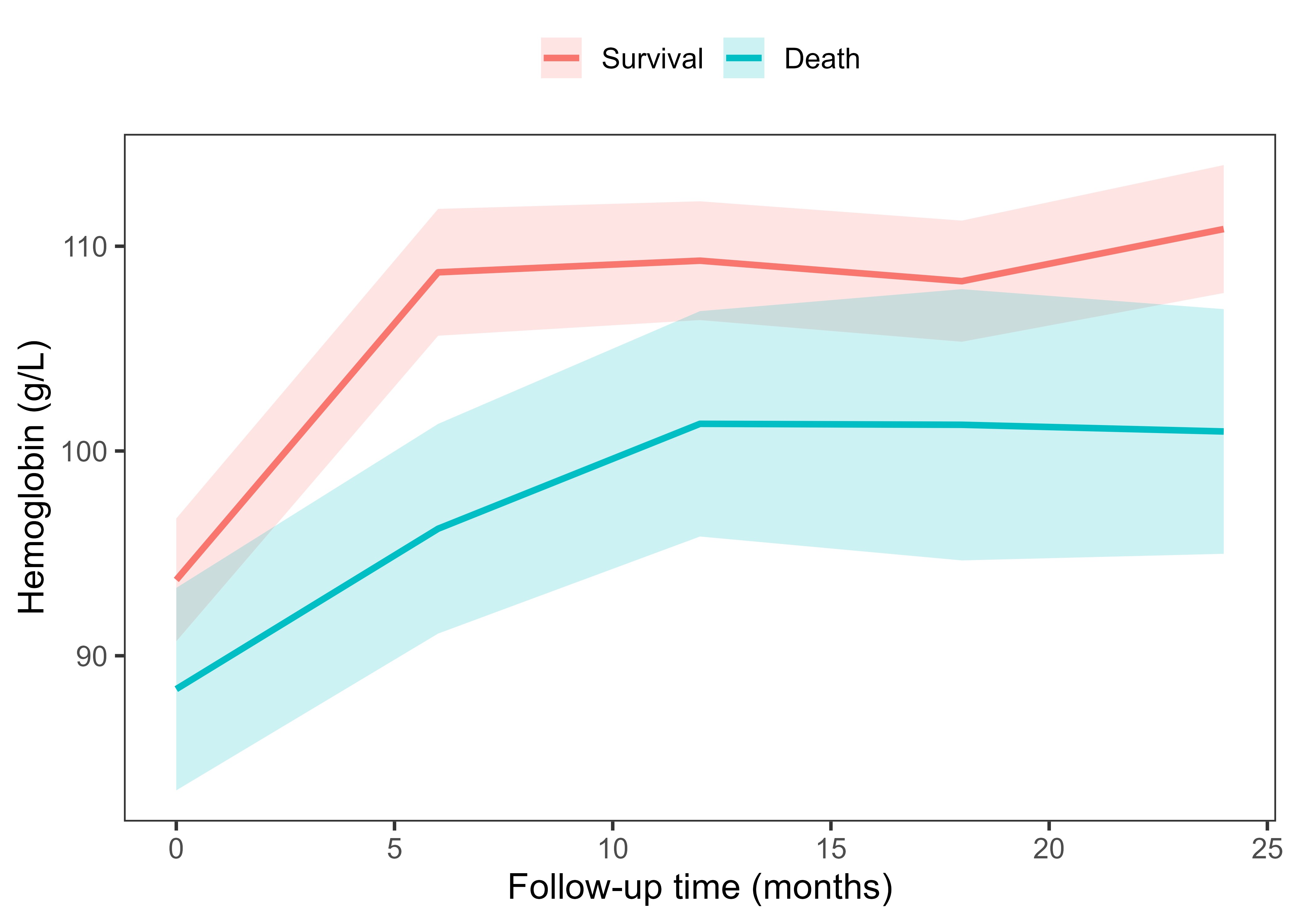

Supplement: SUPPLEMENTARY FIGURE S2 — Longitudinal hemoglobin trajectories by patient outcome. Mean hemoglobin levels during follow-up are shown for patients who survived and those who died. Shaded areas represent 95% confidence intervals. [file Image_2.JPEG]

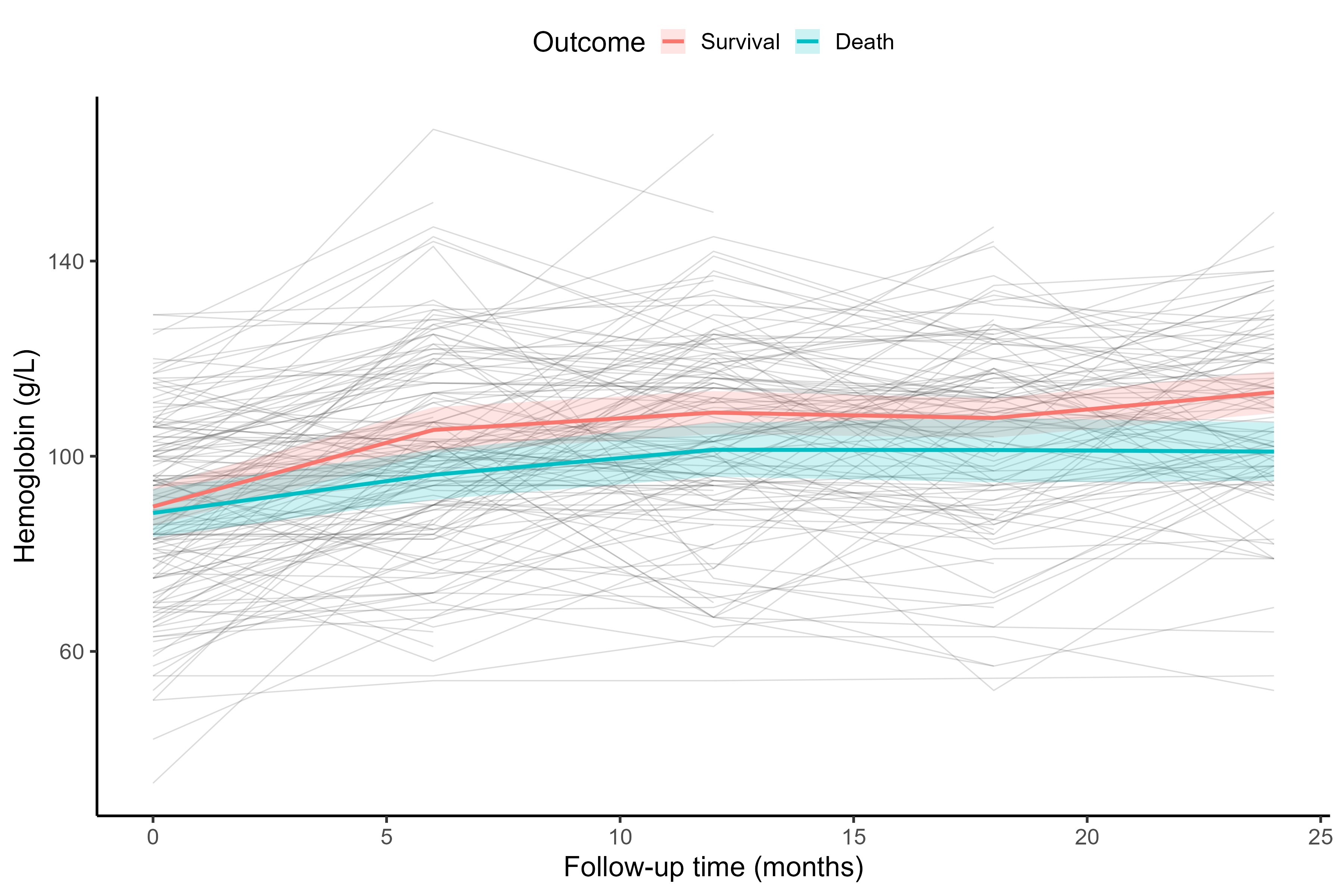

Supplement: SUPPLEMENTARY FIGURE S3 — Individual hemoglobin trajectories by patient outcome. Grey lines represent individual patient hemoglobin trajectories. Solid lines and shaded bands indicate group mean hemoglobin levels and 95% confidence intervals over follow-up. [file Image_3.JPEG]

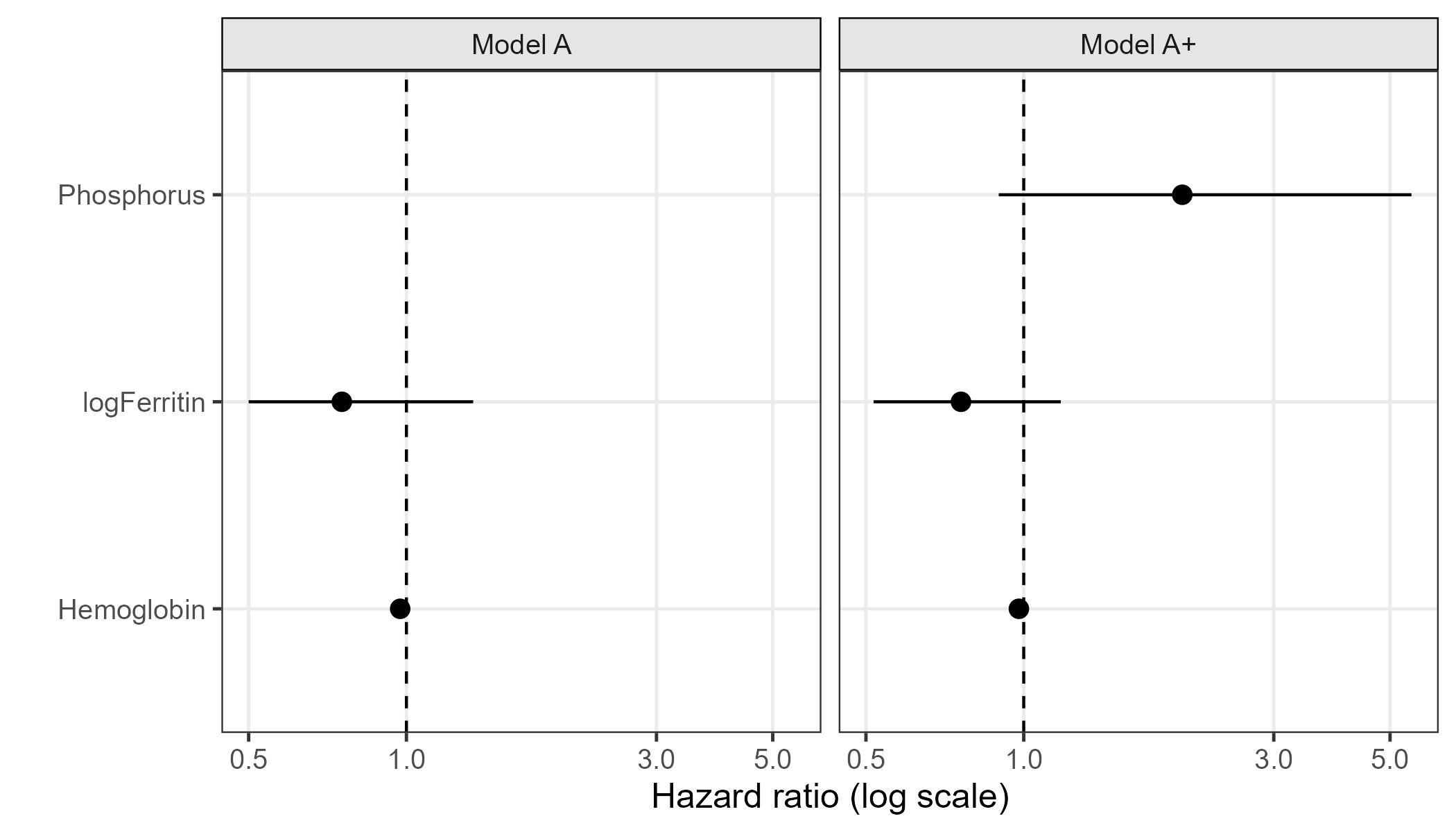

Supplement: SUPPLEMENTARY FIGURE S4 — Sensitivity analysis: forest plot of alternative joint models. Hazard ratios (HRs) for Models A+, A+_winsor, and Model B (TSAT model) demonstrating the robustness of associations across alternative model specifications. [file Image_4.JPEG]
